# Editorial: Essence of survival: impact of primary and secondary metabolism on plant acclimation to abiotic stress

**DOI:** 10.3389/fpls.2025.1651327

**Published:** 2025-08-22

**Authors:** Saad Sulieman, Weiqiang Li, Lam-Son Phan Tran

**Affiliations:** ^1^ Department of Agronomy, Faculty of Agriculture, University of Khartoum, Khartoum North, Sudan; ^2^ Department of Integrative Agriculture, College of Agriculture and Veterinary Medicine, United Arab Emirates University, Al Ain, Abu Dhabi, United Arab Emirates; ^3^ Jilin Da’an Agro-Ecosystem National Observation Research Station, State Key Laboratory of Black Soils Conservation and Utilization, Northeast Institute of Geography and Agroecology, the Chinese Academy of Sciences, Changchun, China; ^4^ Institute of Genomics for Crop Abiotic Stress Tolerance, Texas Tech University, Lubbock, TX, United States

**Keywords:** abiotic stress, climate change, genetic engineering, metabolic pathways, plant adaptation mechanisms, stress alleviation

## 1 Introduction

The most sustainable strategy for feeding the growing population is to intensify research efforts to design new elite genotypes to achieve global food security. Understanding the mechanisms and strategies of plant acclimatization to a dynamic and often hostile environment remains a pressing issue. These priorities are essential given the sessile nature of crop plants and the significant threats related to global climatic conditions. With this in mind, this Research Topic was established to showcase cutting-edge research on plant resilience to abiotic stresses. The central theme is to compile the recent studies deciphering the interplay between primary and secondary metabolism and plant acclimatization to sub-optimal or adverse environmental conditions.

## 2 Mechanisms and strategies of plant acclimatization to abiotic stresses

### 2.1 Metabolic reprogramming of primary metabolism is an indispensable strategy for withstanding abiotic stresses

Several publications have confirmed that cellular metabolism dynamically adjusts in response to adverse conditions in both model and non-model plant species. In an attempt to decipher the essence of survival in *Epimedium pubescens*, Liu et al. emphasized that precision nitrogen (N) fertilization management is crucial for optimizing plant growth and conserving the biosynthesis of specialized secondary metabolites that define the qualitative aspects of this non-model medicinal species (trade-off of resources). By adopting a combination of integrative transcriptomic, physiological, and biochemical analyses, the authors identified a set of key enzymes and N-responsive genes that were coordinately linked to whole-plant growth, carbohydrate metabolism and secondary metabolism.

### 2.2 Secondary metabolism: a vital strategy for augmenting plant resilience to abiotic stress

Research efforts have recently intensified to decipher the biosynthesis and kinetic mode of action of specialized metabolites characterized in various plant species under stressful conditions. Muthusamy and Lee contributed to this field by comprehensively reviewing the recent research documented on the genus *Brassica* to decode the genetic basis of stress-responsive secondary metabolites, with a focus on their regulatory networks. This could pave the way for the development of stress-resilient crops. This review and similar published reports with other focal genera show that specialized metabolites serve as key mediators and stress-relieving compounds, especially against oxidative stress (i.e., reactive oxygen species) induced in response to environmental cues. The authors claim that some secondary metabolites may also serve as biochemical shields and potential signals that promote and stabilize the plant’s cellular machinery (e.g., osmotic regulation and membrane stabilization) during abiotic stresses.

Wan et al. explored the impact of selenium nanoparticles (SeNPs) on plant secondary metabolism using *Polygonatum kingianum* coll. et Hemsl as a test plant. By adopting a multi-omics approach, the authors provided molecular insights elucidating the regulatory networks that control plant responses upon imposition of SeNPs. In particular, the study revealed a set of genes and numerous metabolic pathways (e.g., polysaccharides, saponins, sesquiterpenoids, and triterpenoids) significantly affected by SeNP applications. Notably, exogenous SeNPs modulate the plant’s metabolic activity, leading to higher polysaccharide and saponin biosynthesis, while concurrently reducing the pool size of flavonoids in SeNP-treated plants. This implies that SeNPs resulted in a trade-off of resources, leading to a negative correlation between flavonoids and polysaccharides and saponins in terms of their biosynthesis. Additionally, by modulating the concentration of exogenous SeNPs, other closely related secondary metabolic pathways, such as the phenylpropanoid one, were reported to precisely control the level of flavonoid biosynthesis.

### 2.3 Complex molecular regulation and metabolic cross-talk define plant tolerance to abiotic stress

Developing next-generation crop varieties with better acclimatization to stressful events could profoundly improve agricultural sustainability (Ngongolo and Mmbando, 2024; Li et al., 2025). Following this approach, Chen et al. focused on the cloning and functional characterization of *OsDUF868.12* (belonging to the *OsDUF868* gene family) by applying various state-of-the-art molecular, biochemical, and physiological techniques. Their main target was to enhance the potential of rice plants to tolerate salt stress. Based on their analyses, *OsDUF868.12* was primarily localized in the cell membrane and exhibited a higher expression in response to salt and cold stresses. The overexpression of this gene consequently promoted rice salt tolerance, while its knockdown showed a reversible trend. The authors claim that the *OsDUF868.12* gene could be a vital genetic target for bioengineering programs to enhance rice tolerance to salinity and other abiotic stresses.

In another study, Liu et al. investigated the cold-adaptive perennial herbaceous plant *Heracleum moellendorffii* Hance via extensive transcriptome and non-targeted metabolome analyses. By constructing a high-quality transcript isoform library and performing comparative genomic analyses on leaf samples, the authors aimed to lay the foundation for functional genomics studies and provide advanced knowledge of this plant species, for which a reference genome has yet to be established. Following the identification of differentially expressed genes and differentially accumulated metabolites, advanced multi-omics analyses revealed that flavonoid biosynthesis in this plant species is associated with their cold tolerance. Correlation analyses also showed a close relationship between transcription factors and some genes that code for proteins, particularly within the flavonoid biosynthetic route.

### 2.4 Strategies for harnessing the development of next-generation adaptive crop varieties

Following their previous attempts to promote crop tolerance to adverse stressful events using low-cost, practical approaches, Hassan et al. have conducted a further study using metabolome assays to characterize the contribution of diethyl aminoethyl hexanoate (DA-6), a plant growth regulator, to improving the tolerance of water-stressed white clover (*Trifolium repens*) leaves. DA-6-exogenous application resulted in a profound reprogramming of numerous leaf metabolic pathways (e.g., those related to photosynthesis, oxidative stress responses, and cell membrane stability), resulting in a higher drought tolerance under water-limited conditions. By unlocking the metabolite profile, it was evident that the foliar spray of DA-6 promoted the pool size of key metabolites (e.g., organic acids, amino acids, and sugar alcohols) which could help sustain white clover’s acclimatization to water deficits by boosting cell membrane stability, photosynthetic efficiency, water balance and resistance to oxidative damage.

## 3 Concluding remarks

We believe that this Research Topic is timely and provides significant advances that could contribute to the ongoing efforts to promote our mechanistic and conceptual understanding of how plants acclimate to abiotic stress in the challenging decades ahead. Future studies should focus on unraveling the complex intrinsic regulatory mechanisms that coordinate and fine-tune homeostasis between primary and secondary metabolism during stressful events, especially under field conditions where multiple stress events occur concurrently. Furthermore, deciphering the molecular codes that control metabolic flexibility in a broad range of plant genotypes that endure abiotic stress resilience remains of great potential value. Other promising research avenues encompass how plant-specific metabolites interact synergistically in response to external cues and how such metabolic cross-talk can be employed efficiently to design stress-tolerant crops with higher productivity.
